# Associated Factors for Prostate Enlargement in Chinese Adult Men Aged <40 Receiving Checkups

**DOI:** 10.1155/2022/4792451

**Published:** 2022-08-11

**Authors:** Xiaoma Zhang, Li Xiao, Li Zhang, Jun Zhou, Zongyao Hao, Cheng Yang, Chaozhao Liang

**Affiliations:** ^1^Department of Urology, The First Affiliated Hospital of Anhui Medical University, Hefei 230022, China; ^2^Department of Urology, The Fourth Affiliated Hospital of Anhui Medical University, Hefei 230012, China; ^3^Department of Health Management Center, The Fourth Affiliated Hospital of Anhui Medical University, Hefei 230012, China

## Abstract

**Purpose:**

Prostate enlargement (PE) is an increase in prostate volume in morphology. PE was also observed in some patients aged <40 with chronic prostatitis. This study aimed to explore the associated factors for PE in Chinese adult men aged <40.

**Methods:**

The medical records of 1851 consecutive Chinese adult men aged <40 in a single center were retrospectively analyzed. The checkup indicator characteristics between the PE and non-PE groups were compared by univariate analysis, and the associated factors were analyzed by multivariate analysis.

**Results:**

The overall prevalence of PE (defined as prostate volume ≥ 20 ml) in adult men aged < 40 was 10.4%. Age and the proportions of subjects with prostate calcification or hypertension were different between the PE and non-PE groups (*P* < 0.05). Multivariate logistic analysis showed that prostate calcification (odds ratio [OR], 1.831; 95% confidence interval [CI], 1.281–2.619; *P*=0.001), hypertension (OR, 1.528; 95% CI, 1.125–2.076; *P*=0.007), and age (OR, 1.117; 95% CI, 1.078–1.159; *P* < 0.001) were associated factors for PE in adult men aged <40.

**Conclusions:**

The prevalence of PE in Chinese adult check-up men aged <40 was not rare. In addition to age, prostate calcification and hypertension were associated factors for PE in Chinese adult men aged <40.

## 1. Introduction

Benign prostatic enlargement (BPE) traditionally describes benign prostatic hyperplasia (BPH) in men aged ≥40. Approximately 50% of men with BPE have lower urinary tract symptoms (LUTS) [1]. Early autopsy studies found that the normal prostate weight of men aged 21–30 was approximately 20 g, and histological BPH can occur at the age of 40 [2]. The volume of the normal prostate in Chinese men aged 20–30 was approximately 20 ml, and the specific gravity of the prostate was close to one [3]. Taking normal prostate volume (PV) as the cutoff, prostate enlargement (PE) was also observed in some chronic prostatitis (CP) patients aged < 40 with LUTS, with PV between 12 ml and 33 ml or of mean (39.7 ± 4.4) ml [4, 5], and it induced by CP should be theoretically included in the term “BPE.” However, the associated factors for PE in adult men aged < 40 are unclear and rarely reported. A retrospective analysis of the medical records of the first checkups of a large number of Chinese adult men was conducted to explore the associated factors for PE in adult men aged < 40 and to offer some references for further study on the association and mechanism between PE and chronic inflammation of the prostate in young Chinese adult men.

## 2. Materials and Methods

### 2.1. Study Subjects

The study was conducted in accordance with the Declaration of Helsinki (as revised in 2013). The ethics committee of the Fourth Affiliated Hospital of Anhui Medical University approved the study (No : PJ-YX2021-020) and waived the informed consent for this retrospective analysis. In this retrospective, observational cross-sectional study, data from the health management database between October 2018 and June 2021 were used to identify 6459 consecutive Chinese men aged 18–89 with a detailed medical history and first checkup data.

The inclusion criteria were as follows: (a) men aged 18–39; (b) complete demographic characteristics, blood, and urine testing records; (c) ultrasonic examination records of the abdomen and urinary system, including the prostate. The exclusion criteria were as follows: (a) insufficientincomplete checkup items; (b) lack of ultrasonography of the urinary system, including the prostate; (c) abnormal prostatic nodules detected by ultrasonography; (d) obvious abnormalities of other tumor markers; (e) malignant tumor; (f) diseases of gonadal axis organs and the use of 5-*α* reductase inhibitors, sex hormones, and other drugs that might affect the volume of the prostate; (g) long-term use of drugs that might affect the evaluation of metabolic syndrome (MS), such as drugs might affect serum lipids; (h) moderate or high renal insufficiency that might affect the levels of blood biochemical metabolites, with the estimated glomerular filtration rate (EGFR) < 60 ml min^−1^ 1.73^−1^ m^−2^. In the current routine physical examinations in China, male tumor markers and transabdominal ultrasonography of the abdomen and urinary system, including the prostate, are usually performed on men aged ≥40. However, some adult men aged <40 also choose to do these items.

### 2.2. Data Collection

This study extracted and recorded the demographic and clinical characteristics, history, medication history, and other data of each subject. The medical staff trained by the health management center collected and recorded the age, height, weight, body mass index (BMI), and blood pressure of all subjects. 20 ml of morning fasting venous blood and 10 ml of urine were collected for the detection of routine blood, routine urine, and blood biochemistry. All subjects included in the analysis underwent transabdominal ultrasound (TAUS) examination (completed by experienced ultrasound professionals) of the abdomen and the urinary system, including the prostate.

### 2.3. Definition of Diagnostic Criteria and Indicators

The ellipsoid formula (longitudinal diameter × transverse diameter × anteroposterior diameter × *π*/6) was used to calculate the PV [6]. Three diameters of the prostate and prostate calcification were measured and recorded by ultrasound doctors. It was reported that when the bladder volume was less than 400 ml, the PV measured by TAUS was consistent with that measured by transrectal ultrasound [7]. Prostate calcification is a hyperechoic focus in the prostate, regardless of its size or location, with or without a posterior acoustic shadow [8]. The EGFR was calculated by the Chronic Kidney Disease Epidemiology Collaboration formula (2009 Edition) [9].

PE in our study was defined as PV ≥ 20 ml based on autopsy and population studies [2, 3, 10]. This 20 ml volume cutoff was also larger than the 75th percentile of all PV measures for the age cohort in our study population. Studies have reported that PE was defined as PV larger than the 75th percentile for age decade, which was another criterion for defining PE, and while there was no unified standard for the definition of PE, the risk of adverse events such as urinary retention in men with PV greater than the 75th percentile increased [11, 12]. In our study, the 75th percentile of PV in men aged 18–29 and 30–39 was 15.07 ml and 16.08 ml, respectively. The diagnostic criteria for MS in China are as follows [13]: (a) Obesity: BMI ≥ 28 kg m−2 or waist circumference  ≥ 90 cm in males; Overweight: BMI ≥ 25 kg m−2; (b) Hyperglycemia: fasting blood glucose ≥ 6.1 mmol l−1 or blood glucose (2 h after glucose load)  ≥ 7.8 mmol l^−1^ and/or diagnosed as diabetes mellitus and treated with hypoglycemic drugs; (c) Hypertension: blood pressure ≥ 130/85 mmHg and/or diagnosed with hypertension and treated with antihypertensive medications; (d) Fasting serum triglyceride (TG) ≥1.70 mmol l^−1^; (e) Fasting serum high-density lipoprotein cholesterol (HDLc) < 1.04 mmol l^−1^. The MS was diagnosed as having three or more items.

### 2.4. Statistical Analysis

Continuous variables with a normal distribution were expressed as the mean ± standard deviation ($\bar{x}$ ± Ѕ). A *t*-test was used to compare continuous variables with homogeneous variance; continuous variables with nonnormal distribution were expressed as the median (1^st^–3^rd^ quartiles) (*M* [*Q*_*1*_–*Q*_*3*_]) and compared by the Mann–Whitney *U* test. Categorical variables were expressed as the constituent ratio or rate (%) and compared by the chi-square test. Multivariate logistic regression models were used to assess the associated factors for PE, with results expressed as odds ratios (OR) and 95% confidence intervals (CI). Statistical analyses were performed using SPSS 22.0 (Statistical Package for the Social Sciences, IBM Corporation, NY, USA). All *p* values were two-tailed, and *p* < 0.05 was considered statistically significant.

## 3. Results

**Figure 1:**
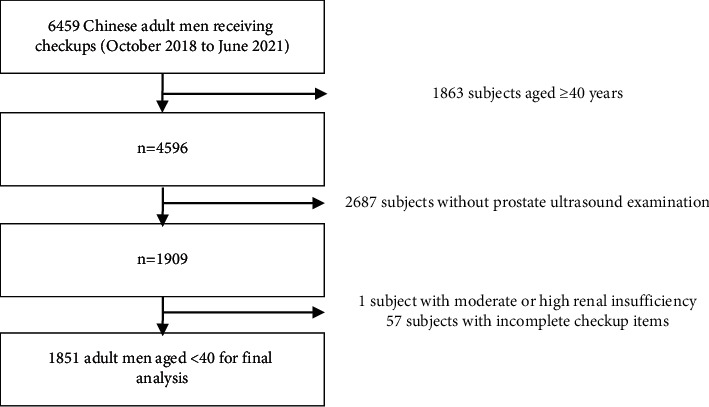
Flow chart of the included subjects.

According to the inclusion and exclusion criteria, 4608 subjects were excluded. A total of 1851 men aged 18–39 were finally included in the analysis (Figure 1). The men aged <40 in the PE group were 20–39 years old; the minimum age of men with PE was 20, with a PV of 24.02 ml. The age range of men aged <40 in the non-PE group was 18–39.

### 3.1. Prevalence of PE in Chinese Adult Checkup Men Aged <40

**Figure 2:**
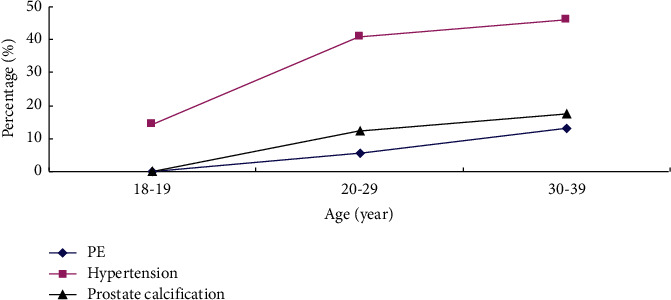
The prevalence of PE, hypertension, and prostate calcification among different ages in adult men aged <40. Abbreviations: PE, prostate enlargement.

The total prevalence of PE in adult men aged <40 was 10.4% (192/1851). The subjects were divided into different age groups according to the age decade to observe the different prevalence of PE among different age groups. The prevalence of PE varied with age groups and increased with age (trend *χ*^2^ = 25.717, *P* < 0.001), as shown in Figure 2. In addition, the prevalence of prostate calcification and hypertension also varied and increased with age (trend *χ*^2^ = 9.577, *P*=0.002; *χ*^2^ = 5.614, *P*=0.018) (Figure 2).

### 3.2. Comparison of Indicator Characteristics between the PE and Non-PE Groups in Chinese Adult Men Aged <40

Univariate analysis showed that the proportions of subjects with prostate calcification and hypertension were different between the PE and non-PE groups (*P* < 0.05) (Table 1). Age, blood pressure, serum total cholesterol, and others were different between the two groups (*P* < 0.05) (Table 2).

### 3.3. Analysis of Associated Factors for PE in Chinese Adult Checkup Men Aged <40

On the basis of univariate analysis, multivariate logistic analysis was carried out with the stepwise forward method under the introduced variables and excluded variables levels of 0.05 and 0.10. Multivariate logistic regression analysis showed that prostate calcification, hypertension, and age were associated factors for PE in Chinese adult men aged <40 (*P* < 0.05) (Table 3).

## 4. Discussion

Histological BPH can lead to morphological BPE. Without histological confirmation, the term “BPE” is preferable to “BPH” in describing PE in men aged ≥40 [1]. Benign PE in men aged ≥40 is mainly caused by BPH, but having benign PE does not necessarily mean BPH. Benign PE was also found in and associated with CP patients aged <40 [4, 5] and its etiology, prevalence, and associated factors in this age range are unknown. At present, studies on the prevalence and risk factors for benign PE are mainly aimed at men aged ≥40. Therefore, it is necessary to understand the prevalence and associated factors for PE in adult men aged <40 to provide new clues for further investigating the association between PE and chronic inflammation of the prostate, which may be helpful for the early prevention and treatment of PE and CP in young adult men. There is no unified standard for the definition of PE, but the literature reports PV ≥ 20 ml [10], ≥25 ml [14], ≥30 ml [15, 16], or ≥40 ml [17]. The definition of PE in the present study was PV ≥ 20 ml, according to autopsy results [2, 3] and population studies [10, 11].

The research results on the prevalence and risk factors for benign PE with LUTS reported by different countries were inconsistent. In addition to regional and ethnic differences, it was related to the nonuniform judgment criteria for PE and the age range of the selected subjects. Garraway et al. [10] reported that the prevalence of benign PE with LUTS (defined as PV ≥ 20 ml) among 699 community men aged 40–79 was 25.3%. Parsons et al. [12] measured PV in 422 subjects aged 27–84 by magnetic resonance imaging and found that 91 subjects (21.6%) had benign PE (PV ≥ 40 ml) on the first visit. The diagnostic rate of benign PE among 1902 Italian men aged >18 increased from 9.3% at the age of ≤50 to 58.7% at the age of >60 in a study based on a digital rectal diagnosis by urologists [18]. Benign PE in those two studies was diagnosed by PV ≥ 40 ml or digital rectal examination, which was inconsistent with the previous autopsy results. Therefore, there are fewer reports on the prevalence of PE in adult men aged <40.

The study results of risk factors for benign PE in men aged ≥40 were also inconsistent. A correlation study between MS, components of MS, and PV in 379 benign PE patients with LUTS aged >50 undergoing surgery indicated that PV was related to systolic blood pressure, serum TG, and serum HDLc levels [19]. A controlled study of 265 benign PE patients with LUTS in their 50s and 248 age-matched healthy people without LUTS demonstrated that PV was negatively correlated with HDLc and positively correlated with low-density lipoprotein cholesterol (LDLc) and total cholesterol (TC) [17]. Parsons et al. [20] documented the research results of 259 benign PE with LUTS patients aged ≥60 and 272 patients aged ≥60 without benign PE in the community. There was no correlation between TC, HDLc, TG, or the TG/HDLc ratio and risk factors for benign PE with LUTS. Park et al. [15] determined that 262 men aged >40 without benign PE with LUTS in the initial examination developed benign PE with LUTS during the 5-year follow-up. Multivariate analysis showed that fat mass and LDLc were important factors for reflecting the occurrence of benign PE with LUTS within 5 years.

Currently, most studies on risk factors for benign PE mainly focus on men aged ≥40. The associated factors for benign PE in adult men aged <40 are rarely reported. The sample size of the present study was large and covered most industries. The results demonstrated that, in addition to age, prostate calcification and hypertension were associated factors for benign PE in adult men aged <40, while MS and dyslipidemia were not associated factors, which was inconsistent with the risk factors for benign PE in men aged ≥40 reported by other scholars.

The mechanism of prostate calcification, hypertension, and age as associated factors for PE in adult men aged <40 is unknown. It is speculated that it may be related to chronic inflammation of the prostate caused by tissue calcification, arteriosclerosis, and the imbalance of sex hormones in the prostate. Prostate calcification affected the symptoms and efficacy of CP [21], interacted with prostate inflammation [22], and was related to age and PV [23]. Hypertension is not only a risk factor for arteriosclerosis and calcification [24] but also a factor affecting PV [25]. It was reported that the chronic inflammation caused by local arteriosclerosis of the prostate artery was also associated with benign PE. The possible mechanism was that lectin-like oxidized low-density lipoprotein receptor-1 induced macrophage infiltration and led to benign PE [26]. It is recognized that benign PE in men aged ≥40 is mainly caused by BPH. BPH mostly occurred in middle-aged and elderly people whose testosterone levels gradually decreased with age. The imbalance of sex hormones may be one of the important causes of BPH in men aged ≥40 [27]. In adult men aged <40 years, the imbalance of sex hormones may be related to chronic inflammation of the prostate. Testosterone plays a regulatory role in cellular signaling and inflammation of the whole immune system by binding to the androgen receptor protein expressed by inflammatory immune cells [28]. Our previous study also found that, compared with the healthy control group, the serum testosterone level of CP patients aged <50 decreased, and the estradiol/testosterone ratio and inflammatory factor levels increased [29].

There are some limitations to our study. First, the data of checkups at a single center in the past 3 years may have been subject to selection bias, but the large number of 1851 samples could be a good way to decrease the bias. Second, the serum sex hormone levels and LUTS of the subjects were not detected and incompletely recorded. Therefore, the correlation between the benign PE associated factors, estrogen/androgen ratio, and CP in our study could not be directly evaluated. Third, prostate ultrasounds were performed by several physicians and the interoperator variability in PV measurements may bring some bias, but experienced ultrasound doctors will try to reduce this bias. For these reasons, we need to conduct further multi-center, multi-community, and larger-sample prospective studies.

## 5. Conclusion

The findings of this study showed that the prevalence of PE in adult men aged <40 was not rare, and that, in addition to age, prostate calcification and hypertension were associated factors for PE in Chinese adult checkup men aged <40. This was the first time that prostate calcification was included in the study of benign PE associated factors. The term “BPE” is not only for adults aged >40 but also for adults aged <40. PE in adult men aged <40 should consider the possibility of chronic inflammation of the prostate. In Chinese adult men aged <40 with prostate calcification and/or hypertension, clinicians may need to advise them on the possibility of the increased risk of having PE and CP.

## Figures and Tables

**Table 1:** Comparison of the proportion of subjects under different indicators in the PE and non-PE groups in adult men aged <40.

Variables		PE (*n* = 192)	Non-pe (*n* = 1659)	*χ* ^2^ value	*P* Value
		*N* (%)	*N* (%)		
Age (year)	18–19	0 (0.0)	7 (0.4)	25.753	<0.001
	20–29	37 (19.3)	619 (37.3)		
	30–39	155 (80.7)	1033 (62.3)		
Hyperglycemia (mmol l^−1^)	Yes (≥6.1)	8 (4.2)	61 (3.7)	0.115	0.735
	No (<6.1)	184 (95.8)	1598 (96.3)		
High triglyceride (mmol l^−1^)	Yes (≥1.7)	76 (39.6)	638 (38.5)	0.092	0.761
	No (<1.7)	116 (60.4)	1021 (61.5)		
Low HDLc (mmol l^−1^)	Yes (<1.04)	77 (40.1)	657 (39.6)	0.018	0.893
	No (≥1.04)	115 (59.9)	1002 (60.4)		
Overweight (kg m^−2^)	Yes (BMI ≥ 25)	96 (50.0)	730 (44.0)	2.505	0.113
	No (BMI < 25)	96 (50.0)	929 (56.0)		
Obesity (kg m^−2^)	Yes (BMI ≥ 28)	14 (7.3)	118 (7.1)	0.008	0.927
	No (BMI < 28)	178 (92.7)	1541 (92.9)		
Hypertension (mmHg)	Yes (BP ≥ 130/85)	107 (55.7)	710 (42.8)	11.672	0.001
	No (BP < 130/85)	85 (44.3)	949 (57.2)		
Metabolic syndrome	Yes	41 (21.4)	260 (15.7)	4.080	0.043
	No	151 (78.6)	1399 (84.3)		
Prostate calcification	Yes	50 (26.0)	239 (14.4)	17.682	<0.001
	No	142 (74.0)	1420 (85.6)		

Values are presented as number (%). Abbreviations: PE, prostate enlargement; HDLc, high-density lipoprotein cholesterol; BMI, body mass index.

**Table 2:** Comparison of indicator characteristics between the PE and non-PE groups in Chinese adult men aged <40 [*M* (*Q*_*1*_–*Q*_*3*_)] or ($\bar{x}$ ± Ѕ).

Characteristics	PE (*n* = 192)	Non-PE (*n* = 1659)	*Z/T* value	*P* Value
Age (year)	34.00 (30.00–37.00)	31.00 (28.00–35.00)	−6.591	<0.001
Height (cm)	173.50 (169.50–177.90)	174.00 (170.00–178.00)	−0.677	0.498
Weight (kg)	74.25 (69.60–82.28)	73.70 (67.40–82.20)	−1.417	0.156
Body mass index (kg m^−2^)	24.99 (23.08–27.42)	24.57 (22.51–26.73)	−2.034	0.042
Systolic blood pressure (mmHg)	128.00 (116.00–140.00)	125.00 (116.00–134.00)	−2.297	0.022
Diastolic blood pressure (mmHg)	83.50 (76.00–93.00)	80.00 (73.00–87.00)	−3.778	<0.001
Neutrophil count (×10^9^ l^−1^)	3.51 (2.85–4.18)	3.41 (2.81–4.13)	−0.285	0.776
Percentage of neutrophils (%)	56.25 ± 8.05	56.17 ± 7.50	0.134^a^	0.893
Lymphocyte count (×10^9^ l^−1^)	2.13 (1.82–2.54)	2.10 (1.79–2.52)	−0.316	0.752
Platelet count (×10^9^ l^−1^)	220.00 (190.25–257.00)	221.00 (190.00–252.00)	−0.737	0.461
Platelet crit (%)	0.23 (0.21–0.26)	0.22 (0.20–0.25)	−2.152	0.031
Serum albumin (g l^−1^)	44.55 (42.70–46.73)	45.40 (43.60–48.10)	−3.890	0.000
Blood urea nitrogen (mmol l^−1^)	4.94 (4.15–5.74)	4.97 (4.34–5.77)	−0.461	0.645
Serum creatinine (*μ*mol l^−1^)	70.85 (64.30–76.88)	69.90 (63.30–76.20)	−0.735	0.462
Serum uric acid (*μ*mol l^−1^)	379.00 (328.00–436.00)	387.00 (337.00–436.00)	−1.140	0.254
EGFR (ml min^−1^ 1.73^−1^ m^−2^)	144.54 (124.20–159.03)	141.49 (124.60–164.14)	−0.240	0.811
Fasting blood glucose (mmol l^−1^)	4.86 (4.56–5.23)	4.92 (4.62–5.24)	−1.370	0.171
Serum triglyceride (mmol l^−1^)	1.52 (1.07–2.25)	1.44 (1.02–2.13)	−1.051	0.293
Serum HDLc (mmol l^−1^)	1.08 (0.96–1.30)	1.09 (0.96–1.25)	−0.241	0.809
Serum LDLc (mmol l^−1^)	2.84 (2.39–3.43)	2.81 (2.32–3.32)	−1.088	0.277
Serum total cholesterol (mmol l^−1^)	4.76 (4.32–5.23)	4.58 (4.04–5.15)	−2.664	0.008
Urine pH value	6.50 (6.00–6.50)	6.50 (6.00–6.50)	−0.109	0.914
Urine specific gravity	1.020 (1.015–1.025)	1.020 (1.015–1.025)	−0.508	0.611

Values are presented as median (1st-3rd quartiles) or mean ± standard deviation. ^a^: *T* value was determined by the *t*-test adopted in the continuous variables with homogeneous variance and normal distribution expressed as the mean ± standard deviation in two groups. Abbreviations: PE, prostate enlargement; EGFR, estimated glomerular filtration rate; HDLc, high-density lipoprotein cholesterol; LDLc, low-density lipoprotein cholesterol.

**Table 3:** Multivariate logistic regression analysis of PE-associated factors in Chinese adult men aged <40.

Associated factors	*β*	SE	Wald *χ*^2^	OR	95% CI for OR		*P* Value
					Lower	Upper	
Constant		−6.062	0.615	97.009	0.002		<0.001
Age (year)	0.111	0.018	36.290	1.117	1.078	1.159	<0.001
Hypertension (mmHg)	0.424	0.156	7.362	1.528	1.125	2.076	0.007
Prostate calcification	0.605	0.182	11.001	1.831	1.281	2.619	0.001

Abbreviations: PE, prostate enlargement; SE, standard error; OR, odds ratio; CI, confidence interval.

## Data Availability

All data generated or analyzed during this study are available from the first author on reasonable request.

## Additional Points

What is already known about this topic? (1). Benign prostatic enlargement (BPE) traditionally describes benign prostatic hyperplasia in men aged ≥40. Approximately 50% of men with BPE have lower urinary tract symptoms. (2). Taking normal prostate volume as the threshold, prostate enlargement (PE) was also observed in some chronic prostatitis (CP) patients aged <40 with LUTS. (3). Correlations between PE and CP and associated factors for PE in adult men aged <40 are unclear and rarely reported. What does this article add? (1). We demonstrated that the prevalence of PE in adult men aged <40 was not rare and that prostate calcification, hypertension, and age were associated factors for PE in Chinese adult checkup men aged <40. (2). We suggested that the term “BPE” was also available for adults aged <40. (3). We provided new clues for further exploring the association between PE and chronic inflammation of the prostate.
